# A New Analyzer Based on Pellistor Sensor with Neural Network Data Postprocessing for Measurement of Hydrocarbons in Lower Explosive Limit Range

**DOI:** 10.1155/JAMMC.2005.55

**Published:** 2005

**Authors:** Radosław Bandomir, Mariusz Krawczyk, Jacek Namieśnik

**Affiliations:** 1 Department of Analytical Chemistry Chemical Faculty Gdańsk University of Technology Gabriela Narutowicza 11/12 Gdańsk 80-952 Poland

## Abstract

We present the results of a first stage of development work on a new type of analyzer for hydrogen and C_1_–C_3_ hydrocarbons concentration measurements in the lower explosive limit range, based on single pellistor sensor with artificial neural network data postprocessing.


Journal of Automated Methods & Management in Chemistry, **2005** (2005), no. 2, 55–57
© 2005 Hindawi Publishing Corporation

### A New Analyzer Based on Pellistor Sensor with Neural Network Data Postprocessing for Measurement of Hydrocarbons in Lower Explosive Limit Range

Radosław Bandomir, Mariusz Krawczyk, and Jacek Namieśnik

*Department of Analytical Chemistry, Chemical Faculty, Gdańsk University of Technology, Gabriela Narutowicza 11/12, 80-952 Gdańsk, Poland*

*Received 5 October 2004; Accepted 26 October 2004*

We present the results of a first stage of development work on a new type of analyzer for hydrogen and $C_1-C_3$ hydrocarbons concentration measurements in the lower explosive limit range, based on single pellistor sensor with artificial neural network data postprocessing.

### 1. INTRODUCTION

In a wide range of industries, especially on drilling platforms and in oil well installations, there is a need for qualitative and quantitative information about various explosive gases. The main interest, in a case of aforementioned installations, lies in main components of natural gas, such as methane, ethane, propane, and butane. For these analyses the most commonly used analytical instruments are simple process chromatographs. Unfortunately such devices have many flaws. They are bulk, costly, they require skilled personnel to operate, and, what is most important, they cannot work in an in-line system. But they have a serious advantage—they are selective. The other instruments used for such type of analyses are different types of sensors—thermoconductive, thermocatalytic, semiconductor, and optical [1, 2, 3, 4, 5, 6, 7, 8, 9, 10, 11, 12]. While in comparison with process chromatographs they are small, cheap, easy to operate and can very well fit in an in-line measurement system, they lack what is an advantage of bigger apparatus—they are in principle nonselective.

A universal sensor, with the possibility of working parameters' regulation, could heighten selectivity achieved by optimization of those parameters for specific analytical tasks. Setting the sensor's working conditions can be made by software means and the program itself can be modified to suit the needs of an analytical process, according to actual knowledge. This kind of sensor can be classified as a new class of sensors—*the software-defined sensor*. A conventional catalytic combustion sensor (pellistor) with power supply and analytic signal circuits controlled by specialized software may be a good example of this new approach to chemical sensors [13].

### 2. EXPERIMENTAL SETUP

Hydrogen (99.999%), methane (99.5%), ethane (99.5%), and propane (99.5%) used for preparation of gaseous mixtures were obtained from Linde AG (Gdańsk, Poland) and were used as received. Air was obtained from the atmosphere with the help of a compressor.

All measurements were conducted using the experimental setup presented in Figure 1, which consists of the following:

(1) a laboratory-made generator of standard gas mixtures;
(2) a laboratory-made measurement cell;
(3) pellistor sensor model OLC20 obtained from Oldham (Dortmund, Germany);
(4) HP 7090A measuring system from Hewlett-Packard (Calif, USA);
(5) a laboratory-made power supply;
(6) an HP 9000/310 instrument controller, data postprocessing was done using a PC with STATISTICA Neural Networks software from Statsoft (Krakow, Poland), http://www.statsoft.pl.

Gaseous mixtures of required concentrations of individual components were prepared by dynamic mixing of pure gas streams in a mixing chamber of generator. The gases were delivered to the mixing chamber from gas cylinders through

Correspondence and reprint requests to Radosław Bandomir, Department of Analytical Chemistry, Chemical Faculty, Gdańsk University of Technology, Gabriela Narutowicza 11/12, 80-952 Gdańsk, Poland; Tel: +48 58 3471527; Fax: +48 58 3472694; E-mail: chemanal@pg.gda.pl.

Table 1: Results obtained from NN for test dataset.

| Dataset number | Hydrogen | | | Sum of methane, ethane, and propane | | |
|---|---|---|---|---|---|---|
| | Known value (% voltage) | Measured value (% voltage) | Deviation (%) | Known value (% voltage) | Measured value (% voltage) | Deviation (%) |
| 1 | 0.9 | 1.1 | 22 | 6.44 | 4.93 | 23 |
| 2 | 1.2 | 0.9 | 25 | 6.90 | 5.14 | 25 |
| 3 | 1.2 | 0.8 | 33 | 5.80 | 5.28 | 9 |
| 4 | 1.5 | 1.0 | 33 | 4.87 | 5.63 | 16 |
| 5 | 1.5 | 0.8 | 47 | 6.70 | 5.22 | 22 |

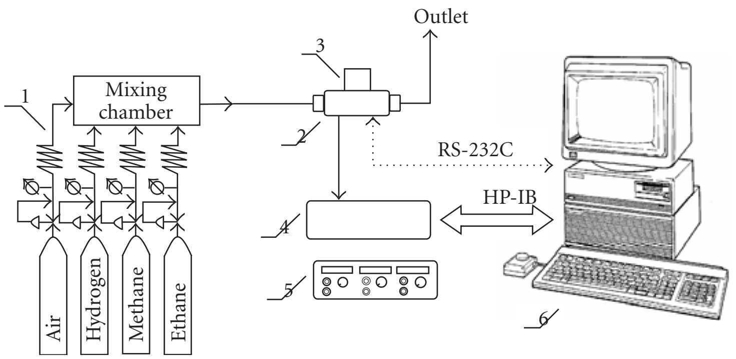

Figure 1: Schematic diagram of experimental setup.

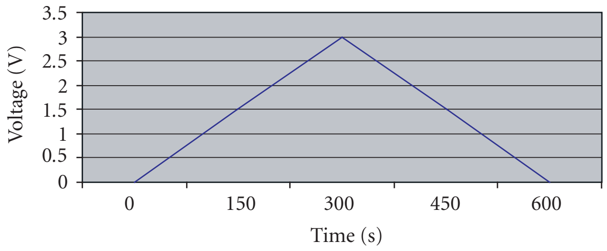

Figure 2: Supply voltage run chart.

a system consisting of pressure regulators and capillaries. In this way a low, stable, and laminar stream of gases was achieved. Flows through each capillary were previously calibrated and flow value to pressure on capillary inlet relation was determined.

The gaseous mixture of known composition from the generator was flowing through the measurement cell with a pellistor sensor mounted. Measurements of pellistor signals were performed in a typical system of an unbalanced Wheatstone bridge. The sensor was supplied by a laboratory-made power supply with a “sawlike” voltage curve increasing from 0 to 3.0 V in a time of 300 seconds, then decreasing in a similar way and so on, as presented in Figure 2.

The voltage arising from destabilization of a bridge as a result of catalytical reaction on the surface of the sensor was measured by a digital voltmeter and recorded by computer as a destabilization voltage versus time chart. A supply voltage versus time chart was also recorded. The data sets obtained this way were then fed to STATISTICA Neural Network software and neural networks were automatically constructed and tested. An equivalent of the sum of concentrations of methane, ethane, and propane was calculated according to (1)

$$\%_{eq} = \%_{methane} + \frac{C_{ethane}}{C_{methane}}\%_{ethane} + \frac{C_{propane}}{C_{methane}}\%_{propane}, \quad (1)$$

where % is a concentration and C is a heat of combustion of corresponding hydrocarbons.

### 3. RESULTS AND DISCUSSION

After supplying of 552 sets of data, each consisting of 300 inputs—destabilization voltage values (1 value per second of supply voltage increase) and 2 outputs—known concentration values (hydrogen and the sum of $C_1-C_3$ hydrocarbons), to ANN generator, the best neural network was found. It had 3 layers: the first consisting of 177 input neurons, the second consisting of 21 hidden neurons, and the third built from 2 output neurons. The results obtained with this network fed with test data set are presented in Table 1 and Figure 3.

As shown in this paper the new analyzer works quite well for this stage of development. It discriminates hydrogen from hydrocarbons, however the discrimination among individual hydrocarbons is still low, hence an equivalent of the sum of hydrocarbons had to be used. The accuracy and precision is quite average and further work, especially finding of better

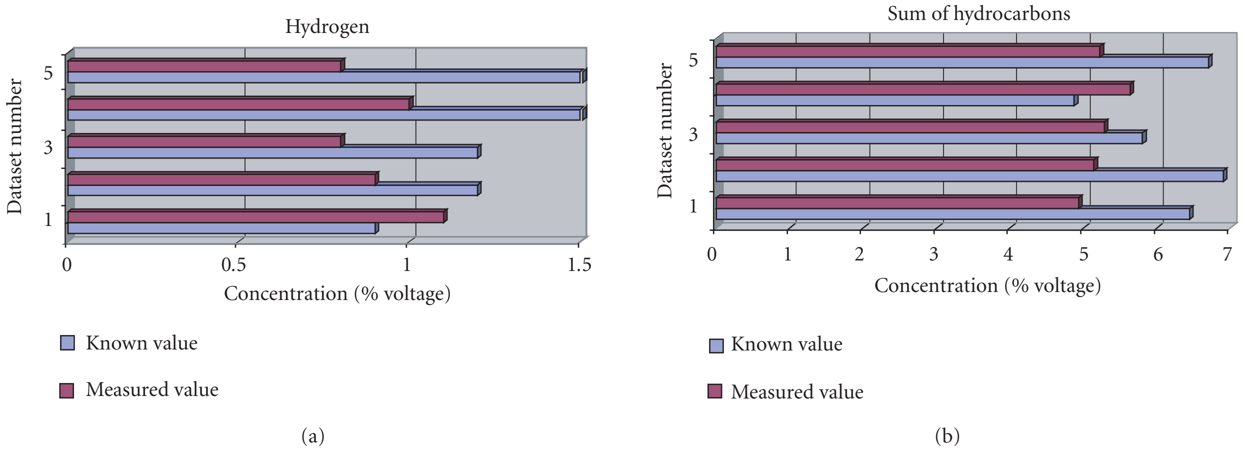

FIGURE 3: Results obtained from NN for test dataset in (a) hydrogen, (b) sum of hydrocarbons.

neural network models, is needed to improve these parameters. A new power supply will be used to allow to precisely manipulate the voltage value and a set of new supplying voltage curves will be tested.

### ACKNOWLEDGMENTS

The Department of Analytical Chemistry constitutes Centre of Excellence in Environmental Analysis and Monitoring which is a research project supported by the European Commission under the Fifth Framework Programme and contributing to the implementation of the Key Action "Sustainable Management and Quality of Water" within the Energy, Environment and Sustainable Development (Contract no EVK1-CT-2002-80010). The authors acknowledge this generous support.

### REFERENCES

[1] Z. Brzóska and W. Wróblewski, *Sensory Chemiczne*, Oficyna Wydawnicza Politechniki Warszawskiej, Warszawa, Poland, 1998.

[2] N. Yamazoe, G. Sakai, and K. Shimanoe, "Oxide semiconductor gas sensors," *Catalysis Surveys from Asia*, vol. 7, no. 1, pp. 63–75, 2003.

[3] S.-D. Choi and B.-K. Min., "$Co_3O_4$-based isobutane sensor operating at low temperatures," *Sensors and Actuators B: Chemical*, vol. 77, no. 1-2, pp. 330–334, 2001.

[4] T. Takada, "A new method for gas identification using a single semiconductor sensor," *Sensors and Actuators B: Chemical*, vol. 52, no. 1-2, pp. 45–52, 1998.

[5] P. T. Walsh and T. A. Jones, "Calorimetric chemical sensors," in *Sensors: A Comprehensive Survey*, W. Gopel, J. Hesse, and J. N. Zemel, Eds., vol. 2, Wiley-VCH, Weinheim, December 1991.

[6] M. Krawczyk, *Nowy Typ Analizatora Do Pomiarów Górnej Granicy Wybuchowoci Mieszaniny Wodoru I Powietrza*, Rozprawa doktorska, Politechnika Gdañska, Poland, 2002.

[7] J. Namieśnik and M. Krawczyk, "Próba zastosowania czujników katalitycznych do ciagłego pomiaru górnej granicy wybuchowości wodoru," *Pomiary-Automatyka-Kontrola*, vol. 5, pp. 15–17, 2001.

[8] M. Krawczyk and J. Namieśnik, "Analizator z czujnikiem katalitycznym do ciagłego monitorowania wybuchowości mieszanin gazowych," *Inż. Aparat. Chem.*, vol. 4, pp. 9–14, 2001.

[9] V. Sommer, P. Tobias, D. Kohl, H. Sundgren, and I. Lundstrom, "Neural networks and abductive networks for chemical sensor signals: a case comparison," *Sensors and Actuators B: Chemical*, vol. 28, no. 3, pp. 217–222, 1995.

[10] H. Debeda, L. Dulau, P. Dondon, F. Menil, C. Lucat, and P. Massok, "Development of a reliable methane detector," *Sensors and Actuators B: Chemical*, vol. 44, no. 1–3, pp. 248–256, 1997.

[11] G. Rose and I. Zdanevitch, "A new method using a catalytic sensor for the identification and concentration measurement of combustible gases," *Sensors and Actuators B: Chemical*, vol. 25, no. 1–3, pp. 426–428, 1995.

[12] B. Chachulski, "Czujniki w badaniach powietrza," in *Materiały Seminarium "Czujniki w Ochronie Środowiska"*, Gdańsk, Poland, 1994.

[13] R. Bandomir, M. Krawczyk, M. Bownik, and J. Namieśnik, "A new type of sensor—software defined sensor—for the selective measurement of hydrogen, methane and ethane in air," in *5th Balaton Symposium on High-Performance Separation Methods*, p. 8, Siofok, Hungary, September 2003.

